# Fitness costs of phage-driven resistance mutations in *Salmonella* Enteritidis populations

**DOI:** 10.1128/jvi.01950-25

**Published:** 2026-01-16

**Authors:** Peilin Lv, Tingting Liu, Siyu Yue, Yu Chen, Yu Wang, Zili Li, Xiue Jin, Yue Li, Xiliang Wang

**Affiliations:** 1 National Key Laboratory of Agricultural Microbiology, College of Veterinary Medicine, Huazhong Agricultural University 627716, Wuhan, China; 2 College of Veterinary Medicine, Huazhong Agricultural University 627716, Wuhan, China; 3 Hubei Provincial Institute of Veterinary Drug Control, Wuhan, China; 4 Hubei Key Laboratory of Animal Nutrition and Feed Science, School of Animal Science and Nutritional Engineering, Wuhan Polytechnic University 554677 , Wuhan, China; Emory University School of Medicine, Atlanta, Georgia, USA

**Keywords:** bacteriophage resistance, evolutionary dynamics, bacterial fitness, *Salmonella *Enteritidis

## Abstract

In the post-antibiotic era, bacteriophages have emerged as viable alternatives for combating antibiotic-resistant infections. Analogous to the emergence of antibiotic resistance, bacteria can also develop resistance to phages, a process often accompanied by a reduction in bacterial fitness. In this study, we investigated the mechanisms behind the development of phage resistance in *Salmonella enterica* serovar Enteritidis strain WJ48 during laboratory coevolution with phage GRNsp8, as well as the associated fitness costs and community dynamics. Three types of phage-resistant mutants, VP81, VP82, and VP84, were isolated. VP81 exhibited a frameshift mutation in the gene encoding glycosyltransferase and displayed partial resistance to GRNsp8. Both VP82 and VP84 carried frameshift mutations in *btuB*, conferring complete resistance to GRNsp8. Deletion of *btuB* abolished phage adsorption and conferred complete resistance. Meanwhile, complementation with *btuB* restored susceptibility. *In vitro* competition assays showed that the *btuB*-deletion strain was competitively disadvantaged relative to the wild-type strain within 24 h, exhibiting a relative fitness value of <1. Consistently, *in vivo* experiments in mice showed a marked attenuation of virulence in the mutant strain. Specifically, the LD₅₀ of the *btuB*-deficient strain in mice was 6.8 × 10⁸ CFU, 121 times higher than the wild-type strain. During the 9-day coevolution experiment, a single-point mutation in *btuB* consistently dominated and represented the primary mode of resistance. These findings shed light on the adaptive trade-offs that bacteria undergo to evade phage infection and provide valuable insights for designing more rational and effective phage therapy strategies that exploit these trade-offs.

**IMPORTANCE:**

As emerging antibacterial agents in the post-antibiotic era, Bacteriophages also face the challenge of bacterial resistance. However, phage resistance development by bacteria is frequently accompanied by a reduction in bacterial fitness. To elucidate the adaptive trade-offs associated with resistance, in this study, we used *Salmonella enterica* serovar Enteritidis strain WJ48 and the broad-host-range bacteriophage GRNsp8 as a model system. We found that the acquisition of phage resistance by bacteria was significantly associated with a reduction in virulence. These findings deepen our understanding of bacteria–phage coevolution but also offer key insights into leveraging the resistance–fitness trade-off to inform the strategic design of more effective phage therapies. The results highlight the potential for improving the application of phages in agriculture and animal husbandry, supporting the sustainable development of phage-based antimicrobial strategies.

## INTRODUCTION

Antibiotics have long been the cornerstone of bacterial infection treatment strategies. Their broad-spectrum efficacy and ease of administration have led to them becoming fundamental to anti-infective therapy (1). In animal husbandry, the use of antibiotics has substantially reduced morbidity and mortality, safeguarding animal health and production efficiency (2, 3). However, widespread and often indiscriminate use has led to the following pressing challenges: (i) the intensification of bacterial resistance with sharply rising mortality from multidrug-resistant infections (4); (ii) residual antibiotics in meat, eggs, and dairy products entering human food chains and threatening public health (2, 5); and (iii) environmental contamination via animal waste, disrupting microbial ecosystems, and amplifying environmental reservoirs of resistance genes (6). Despite global initiatives, such as the World Health Organization’s 2015 Global Action Plan on Antimicrobial Resistance (https://www.who.int/publications/i/item/9789241509763) and integrated One Health strategies, progress has been hindered by persistent antibiotic-dependent farming practices and gaps in policy implementation. The threat posed by the post-antibiotic era compels the exploration of diverse solutions.

Bacteriophages are viruses that infect bacteria, and their abundance in nature is approximately 10 times that of bacteria (7, 8). They are key regulators of microbial communities and are extensively distributed throughout diverse environments, including soil, water, and animal intestines (9–11). To date, over 6,000 bacteriophages have been identified (12), and many of these provide resources for targeting common livestock pathogens, such as *Escherichia coli*, *Salmonella*, and *Staphylococcus aureus*. Bacteriophages attach to host bacterial cells by specifically recognizing receptors on the bacterial surface, such as flagella, pili, and cell wall components (13, 14). Subsequently, they inject their genetic material into the bacterial cell, replicating them extensively using the host’s biosynthetic machinery, and ultimately lyse the bacteria to release progeny phages (15, 16). Owing to their high specificity, evolutionary adaptability, and minimal ecological impact, bacteriophages are considered among the most promising biotherapeutic agents in the modern era (17). However, bacterial resistance remains a major obstacle to developing effective phage therapies, particularly under conditions of single-phage application or prolonged low-dose treatment (18, 19). Although this process of resistance is similar to the selection pressure exerted by antibiotics, the underlying mechanisms differ (20). Thus, understanding how to overcome this resistance and maintain the efficacy of phage therapy has become a central focus of research.

Various intervention strategies can effectively minimize the emergence of bacterial resistance, including (i) using phage cocktails that target diverse bacterial receptors (21–23); (ii) prioritizing the delivery of high-dose phages directly to the site of infection to prevent the development of resistance driven by sub-inhibitory concentrations, a common issue associated with traditional antibiotics (24); (iii) engineering the host range-determining regions of phage tail fiber proteins to expand host specificity and enhance lytic capacity (25); and (iv) integrating phage therapy with antibiotics to synergistically enhance antimicrobial efficacy (26). However, the selective pressure exerted by phages can be leveraged to reshape bacterial populations, increasing their genetic heterogeneity and rendering them more vulnerable to existing therapeutic strategies (27). Although the emergence of phage-resistant bacterial strains is likely inevitable, phage-driven evolutionary pressure often forces bacteria to acquire mutations that facilitate their escape from phage infection for other vital functions. These fitness trade-offs may include impaired nutrient uptake and disruptions to key metabolic pathways (28–30). For example, Chatterjee et al. (31) found that *Enterococcus faecalis* developed phage resistance through non-conservative mutations in the *epa* gene cluster under phage selection pressure. However, these *epa* mutants also exhibited increased susceptibility to antibiotics that target the bacterial cell wall. Similarly, Cai et al. (32) reported that mutations in glycosyltransferase-encoding genes in *Klebsiella pneumoniae* promoted phage resistance in strains with deficient capsule synthesis; however, these resistant strains were more readily phagocytosed by macrophages. Santander and Robeson (33) observed that the loss of the O-antigen in the lipopolysaccharide structure led to phage resistance in *Salmonella enterica* serovar Enteritidis, while also reducing its virulence in *Caenorhabditis elegans*. Leveraging the biology of phage–bacteria coevolution to force bacteria into adaptive trade-offs while developing phage resistance will not only facilitate the repurposing of existing clinical strategies for treating bacterial infections but also promote the sustainable development of phage therapy.

In this study, we investigated the mechanisms underlying the evolution of resistance in bacteria (*S. enterica* serovar Enteritidis strain WJ48) under phage selection pressure (broad-spectrum bacteriophage GRNsp8) and controlled laboratory conditions. We also examined changes in bacterial fitness in phage-resistant mutants. To this end, we designed a 9-day bacterial–phage coevolution experiment, followed by whole-genome sequencing and analysis of phage-resistant mutants isolated at various time points, to identify induced mutations. The findings enhance our understanding of how different genes contribute to phage resistance and the extent to which phage resistance imposes fitness costs on bacteria. These insights will be valuable for developing phage therapies that exploit bacterial evolutionary trade-offs.

## RESULTS

### Mutations in glycosyltransferase and *btuB* genes promote phage resistance

After overnight incubation of bacterial plates with 10 μL of phage stock solution at a concentration of approximately 10⁹ PFU/mL, eight individual colonies emerged within the lysis zones the following day. To identify the genomic alterations associated with phage resistance, all eight colonies underwent whole-genome resequencing and were compared with the WJ48 reference genome. Single-nucleotide polymorphism (SNP) analysis revealed genetic differences between these colonies and the wild-type WJ48 strain, which were primarily grouped into three categories. The corresponding mutants were designated VP81, VP82, and VP84 (Table 1).

**TABLE 1:** Summary of genetic variations in VP81, VP82, and VP84 compared with the reference genome

	Chrom	Pos	Type	Ref	Alt	Ftype	Effect^*a*^	Gene	Product
VP81	Chrom1	1748505	ins	T	TA	CDS	frameshift_variant c.990dupA p.Tyr331fs		Glycosyltransferase family two protein
VP82	Chrom1	4377915	del	TACGCCAGCTG	T	CDS	frameshift_variant c.1347_1356delCAGCTGGCGT p.Ser450fs	*btuB*	TonB-dependent vitamin B12 receptor BtuB
VP84	Chrom1	465853	ins	T	TG				
	Chrom1	4114665	snp	T	C				
	Chrom1	4377659	ins	A	AC	CDS	frameshift_variant c.1612dupG p.Val538fs	*btuB*	TonB-dependent vitamin B12 receptor BtuB

^*a*^ c. and p. denote nucleotide and amino acid changes, respectively, following HGVS nomenclature.

In VP81, a frameshift mutation was identified in the gene encoding glycosyltransferase, which may have altered the polysaccharide structure on the bacterial surface, impairing phage recognition and adsorption, ultimately conferring resistance to the phage. In both VP82 and VP84, mutations were detected in the *btuB* gene. *btuB* encodes a TonB-dependent vitamin B₁₂ receptor, which was confirmed as a phage adsorption receptor by Zhao et al. (34). In VP82 and VP84, the mutations resulted in truncation of the BtuB protein, shortening the polypeptide chain from 614 amino acids to 459 and 547 amino acids, respectively. SWISS-MODEL predictions of the truncated protein structure revealed the location of Ser450 at the C-terminus of β-strand 20, whereas Val538 resides in the middle of β-strand 21. These mutations lead to incomplete β-strands 20 and 21, causing localized collapse of the β-barrel wall (Fig. S1), which may impair vitamin B₁₂ transport efficiency, increase outer membrane permeability, and reduce bacterial viability. In addition, two mutations were identified in the non-coding regions of VP84; however, their potential impact on protein translation is likely minimal compared to that within the coding sequences.

**Fig 1:**
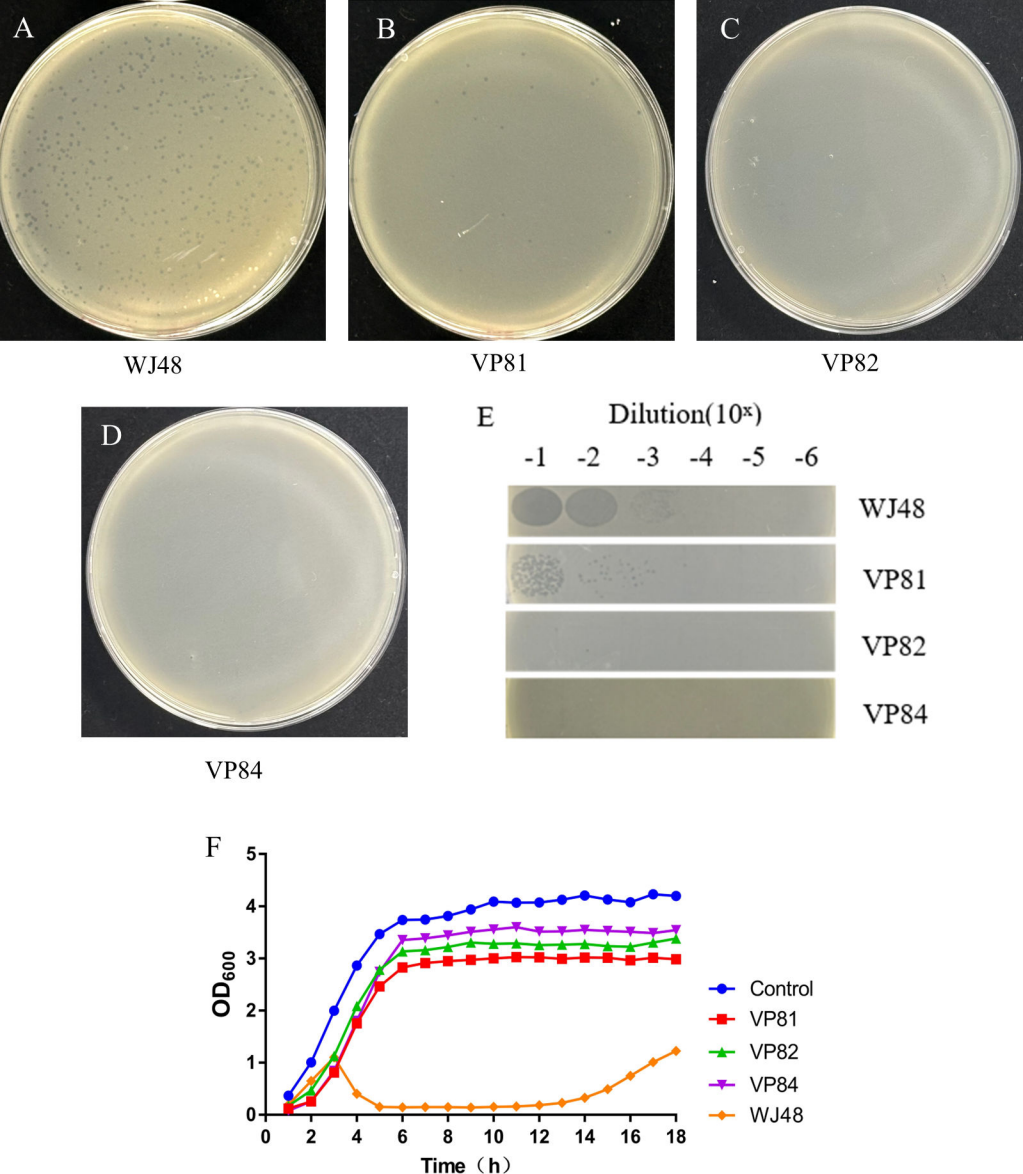
Screening and identification of phage-resistant strains. (**A–D**) The sensitivity of strains WJ48, VP81, VP82, and VP84 to phage GRNsp8 was assessed using the double-layer agar method. (**E**) Effect of mutations on EOP. GRNsp8 (5 µL) at various dilutions was spotted onto LB double-layer agar plates, with the top agar seeded with bacteria at a concentration of 10⁸ CFU. The plates were incubated at 37°C for 24 h, after which plaque formation was evaluated. (**F**) Growth curves of the wild-type strain WJ48 and mutants VP81, VP82, and VP84 in the presence of phage GRNsp8 (MOI = 0.001). Data are presented as mean ± standard deviation (*n* = 3)

Double-layer agar and plaque assays demonstrated that the wild-type WJ48 was lysed by phage GRNsp8 (Fig. 1A). The efficiency of plating (EOP) for the mutant VP81 was reduced to 0.06 (Fig. 1E; Table 2), indicating partial resistance, as it remained susceptible to phage infection (Fig. 1B). In contrast, VP82 (Fig. 1C) and VP84 (Fig. 1D) were resistant to phage infection and showed no susceptibility to GRNsp8. When WJ48, VP81, VP82, and VP84 were co-incubated with the phage, the optical density at 600 nm (OD₆₀₀) value of WJ48 began to decline shortly after entering the exponential growth phase (Fig. 1F), indicating substantial phage-induced lysis. OD₆₀₀ levels began to recover only after 14 h, suggesting the emergence of resistant mutants. In contrast, the growth of VP81, VP82, and VP84 was unaffected by the presence of the phage; following exponential growth, they entered the stationary phase, maintaining relatively stable OD₆₀₀ values throughout. These findings confirmed that VP81, VP82, and VP84 are phage-resistant mutants. Subsequent experiments will focus on the detailed characterization of these three phage-resistant variants. EOP was calculated by dividing the average PFU of the respective mutants by the average PFU of the host bacteria.

**TABLE 2:** GRNsp8 efficiency of plating

Bacteriophage	Efficiency of plating			
	WJ48	VP81	VP82	VP84
GRNsp8	1.00	0.06	0	0

### GRNsp8 exhibits decreased or lost adsorption capacity against resistant strains

**Fig 2:**
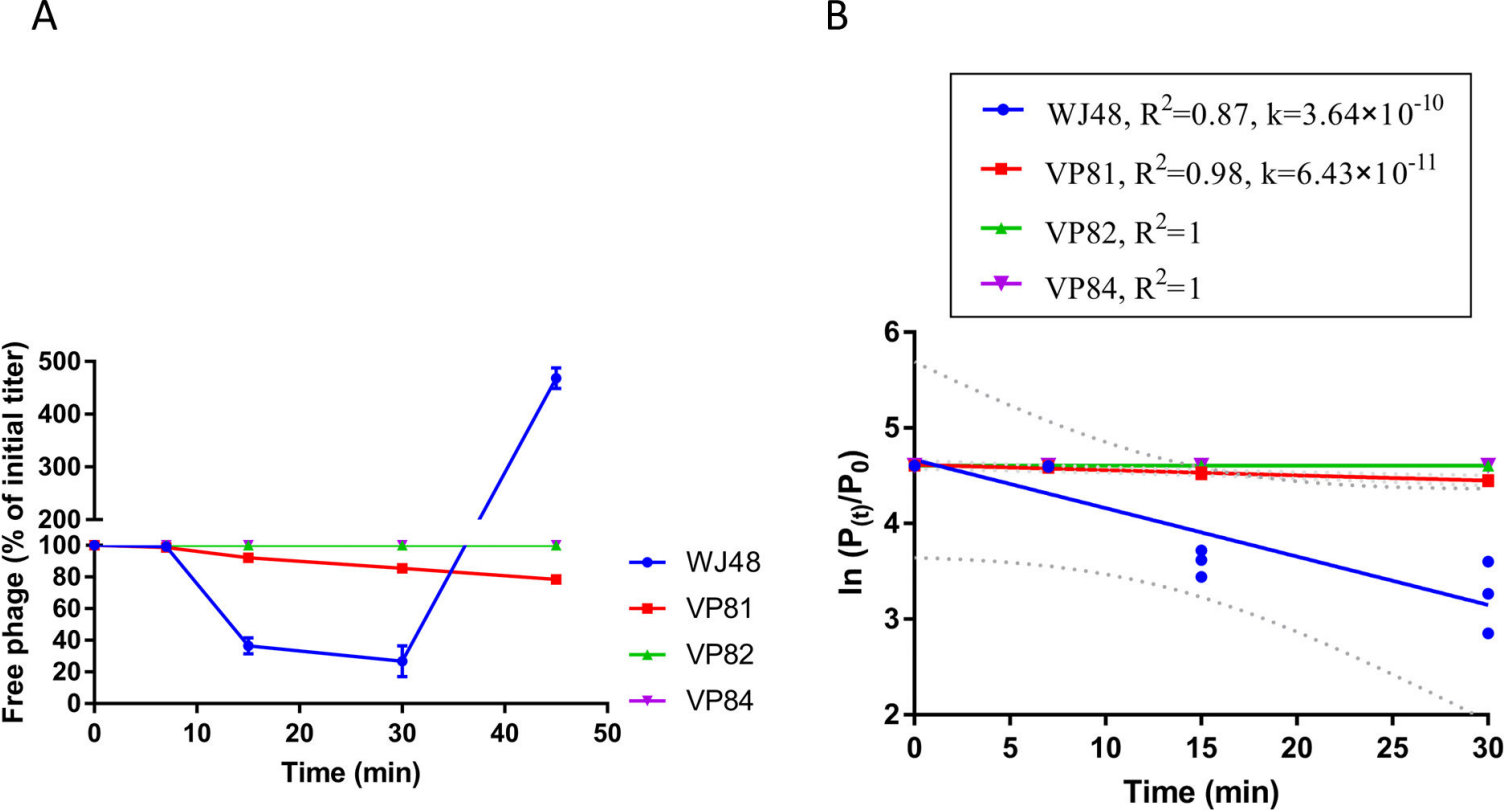
Adsorption kinetics of phage GRNsp8 to strains WJ48, VP81, VP82, and VP84. (**A**) Changes in the percentage of free phage relative to the initial titer over time. Data represent the mean ± standard deviation from three independent experiments. (**B**) Determination of adsorption rate constants (*K*) by linear regression of ln(*Pₜ/P₀*) versus time.

In bacteria, the structure or three-dimensional conformation of surface receptors is commonly altered to prevent phage adsorption. As the identified mutations in both glycosyltransferase and *btuB* genes (via genome sequencing) are associated with phage adsorption, differences in the adsorption kinetics of GRNsp8 against the three classes of resistant strains were determined. As shown in Fig. 2, in the wild-type strain WJ48, the proportion of free phage declined rapidly over time, indicating efficient adsorption. The adsorption rate constant (*k*) was 3.64 × 10⁻¹⁰ mL/min (*R²* = 0.87), reflecting a high phage-binding capacity. In contrast, strain VP81 showed a slower adsorption rate (*k* = 6.43 × 10⁻¹¹ mL/min, *R²* = 0.98). This suggests that the mutation in the glycosyltransferase gene partially impaired phage adsorption. Meanwhile, strains VP82 and VP84, both harboring mutations in *btuB*, showed almost no decrease in free phage titers throughout the 45-min observation period. These findings suggest that the complete resistance observed in VP82 and VP84 results from failed phage adsorption.

### The mutation in *btuB* confers complete resistance to bacteriophage

**Fig 3:**
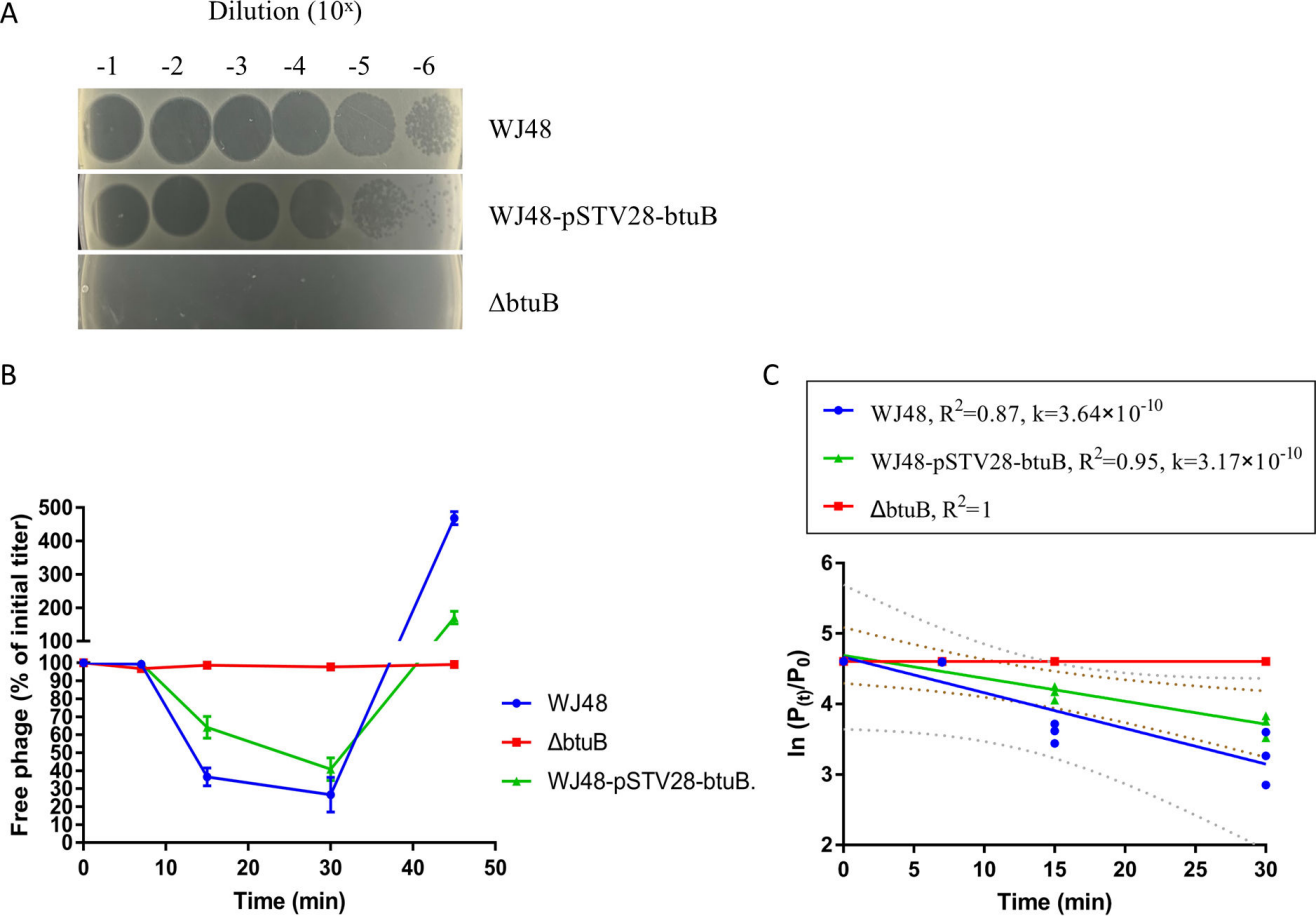
Phage sensitivity and adsorption assays. (**A**) Spot assay of serially diluted phage GRNsp8 on WJ48, WJ48-pSTV28-*btuB*, and the Δ*btuB* mutant. (**B**) Adsorption curves showing the percentage of free phage relative to the initial titer over time. Data represent the mean ± standard deviation from three independent experiments. (**C**) Determination of adsorption rate constants (*K*) by linear regression of ln(*Pₜ/P₀*) versus time.

To determine whether the mutation in *btuB* was responsible for the observed resistance to bacteriophage, we constructed a *btuB*-knockout mutant and assessed its sensitivity to the phage GRNsp8. Spot assays revealed that GRNsp8 formed clear plaques on wild-type WJ48, whereas no lytic activity was observed in the Δ*btuB* mutant. The phage produced similarly clear plaques on the complemented strain WJ48-pSTV28-btuB (Fig. 3A). Adsorption assays further indicated that GRNsp8 failed to adsorb to the Δ*btuB* strain. In the wild-type strain WJ48, the proportion of free phage rapidly decreased to <50% of the initial titer within 15–20 min and reached its lowest level at 30 min. The complemented strain WJ48-pSTV28-btuB showed a similar adsorption pattern, with a decline in free phage in the first 30 min. In contrast, the *ΔbtuB* mutant maintained an almost 100% free phage throughout the assay (Fig. 3B). To quantify these differences, the adsorption rate constants were calculated by fitting ln(*Pₜ*/*P₀*) versus time (Fig. 3C). The wild-type WJ48 had an adsorption rate constant of 3.64 × 10⁻¹⁰ mL/min (*R²* = 0.87). Meanwhile, the complemented strain WJ48-pSTV28-*btuB* had a comparable value of 3.17 × 10⁻¹⁰ mL/min (*R²* = 0.95). In contrast, the *ΔbtuB* strain showed an essentially horizontal regression line (*R²* = 1). These findings confirm that BtuB functions as an adsorption receptor for GRNsp8 and that disrupting BtuB impairs phage binding, conferring complete resistance.

### Acquisition of phage resistance imposes a fitness cost on bacterial populations

**Fig 4:**
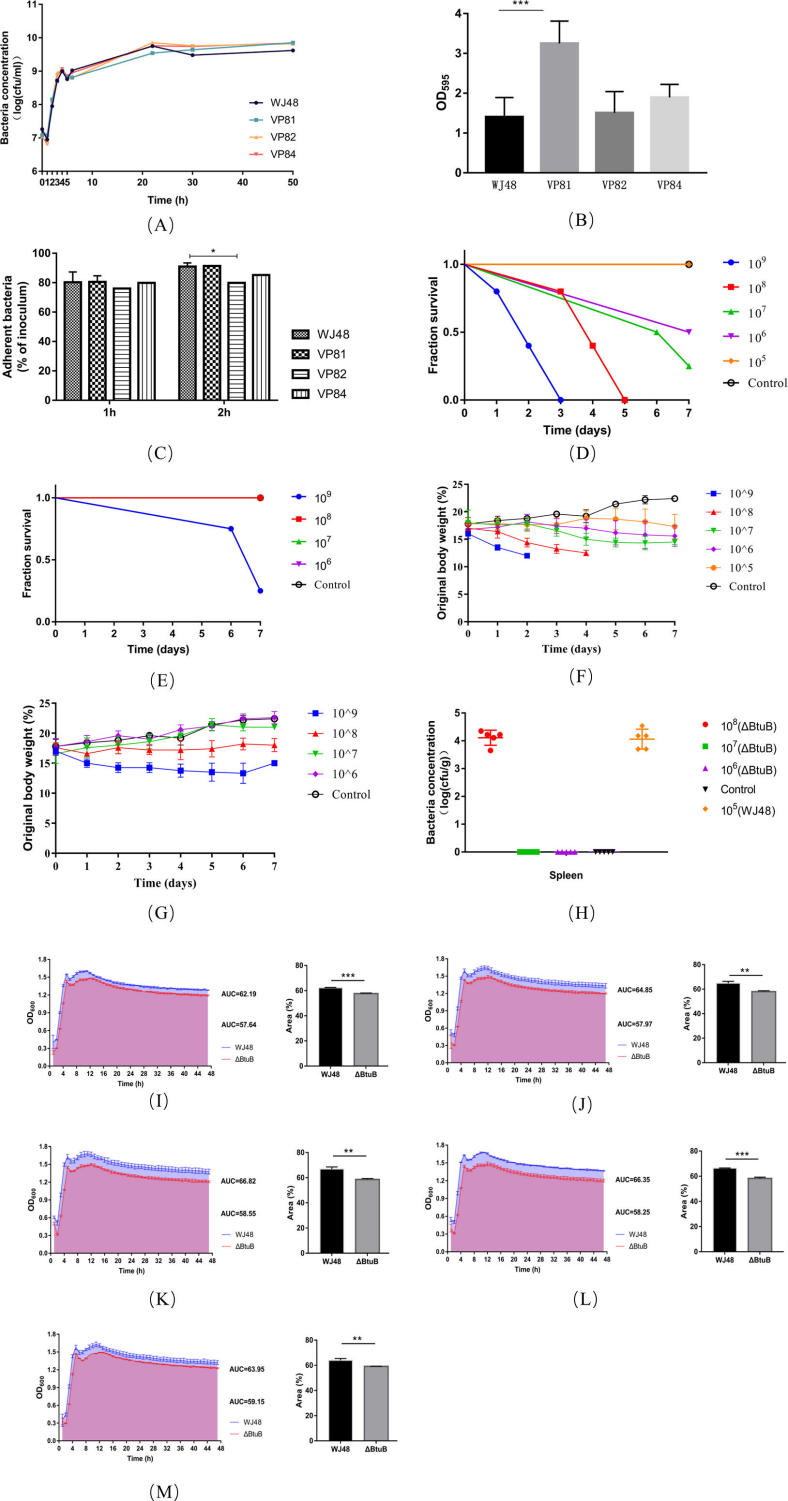
Changes in bacterial fitness. (**A**) Growth dynamics of strains WJ48, VP81, VP82, and VP84 over a 50-h period, expressed as bacterial concentration (CFU/mL). (**B**) Optical density (OD₅₉₅) measurements of the strains. Data are presented as mean ± standard deviation; ***P < 0.001. The assessment of biofilm-forming ability was performed according to the method described by Khunnamwong et al. (36). The OD₅₉₅ value of the negative control group was defined as ODc: OD ≤ ODc: no biofilm formation; ODc < OD ≤ 2× ODc: Weak biofilm formation; 2× ODc < OD ≤ 4× ODc: Moderate biofilm formation; OD > 4× ODc: Strong biofilm formation. (**C**) Adhesion rates of the bacterial strains to CT26 after 1 and 2 h. All experiments were conducted in triplicate, and data are presented as mean ± standard deviation; *P < 0.05. (**D**) survival curves of mice infected with wild-type WJ48 at the respective doses. (**E**) Survival curves of mice infected with the Δ*btuB* mutant at doses of 10⁹, 10⁸, 10⁷, and 10⁶ CFU per mouse. (**F**) Percentage change in body weight of mice infected with wild-type WJ48 at doses of 10⁹, 10⁸, 10⁷, 10⁶, and 10⁵ CFU per mouse. (**G**) Percentage change in body weight of mice infected with the Δ*btuB* mutant at doses of 10⁹, 10⁸, 10⁷, and 10⁶ CFU per mouse. Data are presented as mean ± standard deviation (*n* = 5). (**H**) Bacterial CFU in the spleen of mice after infection with different bacterial doses. Each symbol represents data from an individual mouse, and the bars represent the mean. (**I–M**) Growth curves of WJ48 and Δ*btuB* under varying VB_12_ concentrations. Bacterial strains were cultured in M9 minimal medium without supplementation (**I**) and with the addition of 1 nM (**J**), 10 nM (**K**), 100 nM (**L**), and 1 μM (**M**) of VB_12_. Growth was monitored over time by measuring the optical density at 600 nm (OD₆₀₀), and growth curves were generated accordingly. The AUC was calculated to quantify the total bacterial growth under each condition. Blue indicates WJ48, and red indicates the Δ*btuB* mutant.

Phage selection pressure drives bacterial mutations that confer resistance to phage infection; however, this resistance often incurs fitness costs. These costs may include reduced virulence, increased sensitivity to antibiotics, and diminished colonization capacity (35). To assess the impact of resistance mutations on bacterial fitness, we evaluated the growth, biofilm formation, cell adhesion, virulence, and nutrient uptake of WJ48 and the mutants in Luria-Bertani (LB) medium. No significant differences in growth were observed among the three resistant strains, and their growth profiles were largely comparable to the profile of the wild-type strain in LB medium (Fig. 4A). However, in the *in vitro* competition assay, which was conducted to compare the relative fitness (W) of the *btuB*-deletion strain with that of WJ48 at different time points during co-culture, the *btuB*-deletion strain consistently exhibited lower fitness than WJ48 throughout the 24-h competition period. The relative fitness values of the *btuB*-deletion strain were 0.70 ± 0.058 at 8 h, 0.85 ± 0.031 at 12 h, and 0.91 ± 0.075 at 24 h, all of which were below the reference value of 1.0 (Table 3).

**TABLE 3:** Relative fitness of the *ΔbtuB* strain compared with the wild-type strain at different time points

Time (h)	Relative fitness (mean ± SD)
8	0.7 ± 0.058
12	0.85 ± 0.031
24	0.91 ± 0.075

Regarding biofilm formation, VP82 and VP84 exhibited weak biofilm-producing capacity similar to that of WJ48. VP81 produced significantly more biofilms than WJ48 (*P <* 0.001), classifying it as a strain with moderate biofilm-forming ability (Fig. 4B). Adhesion assays showed no significant differences in the attachment of WJ48 and the mutants to CT26 after 1 h of infection. However, after 2 h of infection, VP82 showed significantly reduced adhesion to CT26 cells compared with the wild-type strain (*P <* 0.05) (Fig. 4C), suggesting attenuated pathogenicity. This observation was confirmed through *in vivo* experiments.

Regarding bacterial virulence, the median lethal dose (LD₅₀) of WJ48 in mice was 5.6 × 10⁶ CFU, whereas that of the *btuB*-deficient mutant reached 6.8 × 10⁸ CFU (121 times higher) (Table 4). All mice infected with 10⁹ CFU of WJ48 succumbed by day three post-injection, and those in the 10⁸ CFU group died by day 5 post-injection (Fig. 4D). Although mice in the 10⁷, 10⁶, and 10⁵ CFU groups survived, their average body weight remained consistently lower than that of the control group (Fig. 4F). In contrast, mortality in mice infected with the Δ*btuB* strain occurred only in the 10⁹ CFU group (Fig. 4E). All mice in the 10⁸ CFU group survived, although their average body weight was lower than that of the control group mice. Body weight trends in the 10⁷ and 10⁶ CFU groups closely resembled those of the control group (Fig. 4G). On the final day of the experiment, necropsy of the surviving mice showed *Salmonella* only in the spleens of mice in the 10⁸ CFU group infected with the *btuB*-deletion strain. The bacterial load was low and comparable to that observed in the 10⁵ CFU group infected with WJ48 (Fig. 4H). This indicated that although the Δ*btuB* strain was capable of host colonization, it did not induce severe clinical symptoms. Deletion of the *btuB* gene markedly attenuated the virulence of the strain in mice.

**TABLE 4:** Determination of LD₅₀ in mice

Dose (CFU/mouse)	No. of animals		No. of deaths		Mortality rate (%)	
	WJ48	Δ*btuB*	WJ48	Δ*btuB*	WJ48	Δ*btuB*
10^9^	5	5	5	3	100	60
10^8^	5	5	5	0	100	0
10^7^	5	5	3	0	60	0
10^6^	5	5	1	0	20	0
10^5^	5	/^*a*^	0	/	0	/

^*a*^ / denotes no infection was performed for that strain at this dose.

To assess bacterial nutrient uptake, we compared the growth characteristics of the wild-type strain WJ48 and *btuB*-deficient mutant (Δ*btuB*) under conditions with and without exogenous vitamin B₁₂ (VB₁₂) supplementation. The results indicated that, in the absence of VB₁₂, the area under the growth curve (AUC) for Δ*btuB* was significantly lower than that for WJ48 (Fig. 4I). Although the AUC of Δ*btuB* increased with increasing VB₁₂ concentration, it consistently remained significantly lower than that of WJ48 (Fig. 4J through L). At a VB₁₂ concentration of 1 μM, the AUC of WJ48 showed a slight decline; however, it still exceeded that of the Δ*btuB* group (Fig. 4M), suggesting that excessive VB₁₂ may induce feedback inhibition and mild toxicity. WJ48 exhibited superior growth performance relative to Δ*btuB* across all tested VB₁₂ concentrations, with the disparity particularly pronounced at lower concentrations. At higher VB₁₂ levels, a weak nonspecific uptake mechanism or an alternative pathway may partially offset the absence of *btuB*. These findings support the idea that *btuB* plays a role in vitamin B₁₂ transport, which could contribute to bacterial growth under certain conditions.

### Mutations in *btuB* are stably inherited during laboratory coevolution

To further investigate the dynamics of the three classes of mutations during bacterial–phage coevolution, a 9-day experiment was conducted. Culture samples were collected at defined time points (24, 48, 72, and 216 h) from the coevolution system to monitor the mutation frequencies of the three resistance-associated genes. To determine whether the phages underwent evolutionary changes during the 9-day co-culture period, their genomes were isolated at four time points. They were extracted, sequenced, and compared with the reference genome GRNsp8. No mutations were detected in the phages. No mutations in glycosyltransferase genes were detected in the 384 samples analyzed across the four time points, suggesting that mutants harboring this type of mutation were at a competitive disadvantage relative to other mutants and were ultimately eliminated during co-cultivation.

**Fig 5:**
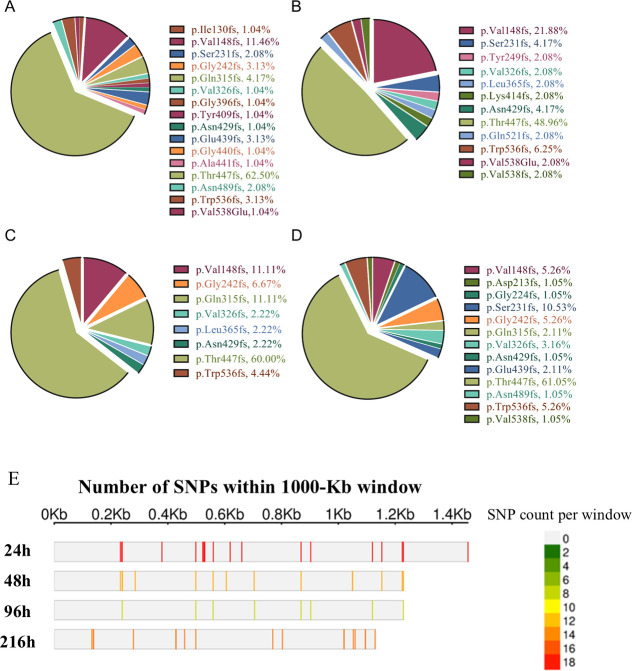
Mutation frequencies in the *btuB* gene among phage-resistant strains. (**A–D**) Proportions of various mutation sites in the *btuB* gene detected at 24 h (**A**), 48 h (**B**), 96 h (**C**), and 216 h (**D**). Different colors represent distinct mutation sites. (**E**) Distribution pattern of SNP density across the *btuB* gene region. The horizontal axis shows the *btuB* gene length (Kb). Each row represents a bacterial isolate at a different time point (h). Colors denote varying SNP densities, with gray indicating the absence of SNPs. The length of the *btuB* gene presented in each row is defined by the position of its last SNP, corresponding to the highest numerical value.

Mutations in the *btuB* gene were prevalent, with observed mutation frequencies of 98.88%, 97.87%, 91.67%, and 96.81% at 24, 48, 72, and 216 h, respectively. A total of 23 single-point mutations were identified, with no evidence of multisite mutations within the same gene. This absence of multisite variation partly indicates that such mutations may carry a higher fitness cost, reduce bacterial viability, and prevent their enrichment in the population. Alternatively, the probability of acquiring multisite mutations within the same gene may be inherently lower than that of a single mutation. Among the single-point mutations, the most frequent was a frameshift affecting threonine 447 of the BtuB protein, occurring at frequencies of 60%, 47%, 54%, and 58% at 24, 48, 72, and 216 h, respectively (Fig. 5A through D). The second most common mutation was a frameshift affecting valine 148, with corresponding frequencies of 11%, 21%, 10%, and 5% (Fig. 5A through D). The apparent mutational preference for these two sites suggests that they may be critical for the structural stability of the BtuB protein (Fig. 5E). In conclusion, mutations in *btuB* were the predominant adaptive mechanism during the 9-day laboratory coevolution experiment, representing the primary strategy by which bacteria acquired resistance to phage infections.

## DISCUSSION

The emergence of bacterial resistance to bacteriophages is a fundamental issue in microbial ecology and in evolutionary biology. As natural predators of bacteria, bacteriophages act as both a source of environmental stress and a selective force in bacterial–phage interactions. The intensity of this selective pressure is a key factor in shaping bacterial evolution. Bacteria have evolved diverse resistance mechanisms to adapt to ever-changing environments and ensure survival. For instance, the CRISPR-Cas system enables bacteria to capture fragments of phage DNA, providing immune memory and facilitating the target elimination of specific viruses (37). Another mechanism involves mutations in the outer membrane protein OmpC, which blocks phage adsorption (38). These mechanisms represent responses to phage infection and the outcomes of the dynamic evolution of microbial communities.

Owing to constraints such as limited resources and the metabolic cost of resistance, bacteria may not always evolve complete resistance to phages. In this study, we isolated the strain VP81, which harbored a frameshift mutation in the gene encoding a glycosyltransferase. Glycosyltransferases play a crucial role in the biosynthesis of bacterial lipopolysaccharides and capsular polysaccharides (39), both of which act as receptors for phage adsorption and recognition on the bacterial cell surface (40–42). VP81 conferred partial resistance to phage GRNsp8 (EOP = 0.06) and was associated with a significant increase in biofilm production. Several factors may contribute to this phenomenon. One possibility is that the mutation induced a bacterial stress response, resulting in the upregulation of other biofilm-associated genes to maintain the adhesion. Another possibility is that VP81 may have adopted a trade-off strategy, mitigating the high energy cost of full resistance while enhancing survival by forming a physical barrier that reduces phage contact, balancing energy expenditure with survival advantage.

Complete resistance is often accompanied by high metabolic costs. For example, the strains VP82 and VP84 harbor frameshift mutations in the *btuB* gene and exhibited full resistance to GRNsp8. BtuB is a TonB-dependent receptor located in the outer membrane of *Salmonella* spp. and is primarily responsible for the uptake of VB₁₂. Within bacterial cells, VB₁₂ serves as a coenzyme in several metabolic pathways. Disruption of BtuB function compromises the bacterium’s ability to efficiently acquire VB₁₂ from the environment, reducing its adaptability and survival. Beyond *Salmonella*, similar phenomena have been observed in other microorganisms. Abellon-Ruiz et al. reported that in gut *Bacteroides*, BtuB homologs form multiprotein outer-membrane complexes together with surface lipoproteins (BtuG). This creates a specialized system capable of efficiently capturing and transporting vitamin B₁₂ in competitive environments (43). In this study, M9 minimal medium (lacking VB₁₂ and its precursors) was supplemented with different concentrations of VB₁₂. Across all tested concentrations, the wild-type strain WJ48 consistently outperformed the Δ*btuB* mutant in terms of growth, with the most significant differences observed under low VB₁₂ conditions. This disparity diminished at higher VB₁₂ concentrations, possibly due to weak nonspecific uptake mechanisms or compensatory pathways that partially offset the limitations imposed by *btuB* deletion. Moreover, both *in vitro* and *in vivo* experiments confirmed that the absence of *btuB* results in decreased bacterial colonization capacity and attenuated virulence in the host.

In the ongoing interaction between bacteria and bacteriophages, a persistent “arms race” drives the differentiation of bacterial populations into multiple subpopulations, each responding to phage pressure distinctly. Although this diversification enhances the overall resilience of the population, the fate of individual subpopulations is determined by the efficacy and metabolic cost of their respective resistance mechanisms. For example, in marine *Aeromonas*, co-mutations in the genes encoding lipopolysaccharide and OMP are retained during coevolution with phages, whereas partial resistance mediated by two-component systems does not significantly accumulate (44). In the present study, mutants with partial resistance mediated by glycosyltransferase were eliminated during a 9-day coevolution experiment, whereas resistant mutants arising from mutations in the *btuB* gene consistently prevailed, establishing *btuB*-mediated resistance as the dominant adaptive strategy. This outcome may be attributed to differences in resource allocation between the resistance mechanisms of the two subpopulations. Glycosyltransferase-mediated resistance likely imposed a competitive disadvantage, resulting in the elimination of inter-subpopulation competition under phage pressure. However, our research on bacteria–phage coevolution has thus far been conducted exclusively under laboratory conditions. These controlled settings, although valuable, are subject to environmental limitations and may not accurately represent the dynamics within a whole organism. *In vivo*, the presence of factors, such as the immune system and metabolic pathways, introduces additional layers of complexity and variability. Therefore, the relevance and complexity of these findings in living organisms require further investigation and validation.

The emergence of bacterial resistance to bacteriophages is shaped by a complex interplay of multifactorial and hierarchical processes. Bacteria-phage coevolution not only enriches our understanding of virus-host dynamics but also serves as a valuable model for investigating microbial adaptability and immune responses. Our findings demonstrate that *btuB*-mediated complete resistance to phages significantly attenuates the virulence of bacteria in the host. Furthermore, this resistant subpopulation maintained genetic stability throughout the coevolution process, underscoring the potential of targeting *btuB*, for example, through inhibitors of vitamin B₁₂ uptake, as a novel strategy for controlling *Salmonella* infections. In conclusion, gaining deeper insights into bacterial–phage coevolution will advance the precision regulation of microbial ecosystems. Harnessing the adaptive costs associated with bacterial resistance may offer new avenues for enhancing the effectiveness of phage therapy in both agriculture and animal husbandry.

## MATERIALS AND METHODS

### Bacterial strains, plasmids, and phages

The bacteriophage GRNsp8 and its host *S. enterica* WJ48 were isolated and preserved in our laboratory for use in this study. The genome of GRNsp8 is a linear double-stranded DNA molecule consisting of 111,357 bp, and its GC content is approximately 39.9%. GRNsp8 belongs to the genus *Epseptimavirus* of the family *Demerecviridae*. The complete genome sequence and detailed annotation of GRNsp8 have been reported by Li et al. (45). The genome of *Salmonella* WJ48 comprises a single chromosome and two plasmids. Plasmid 1 carries the *spv* gene cluster, which plays a crucial role in systemic infection. The pKD4, pKD46, and pCP20 plasmids used for λ-Red homologous recombination were generously provided by the Laboratory of Microbiology and Immunology. The plasmid pSTV28 was obtained from TaKaRa (Beijing, China). The plasmid pBBR1 used for bacterial fluorescence labeling was acquired from the MiaoLing Plasmid Platform (Wuhan, China).

### Isolation and identification of phage-resistant mutants

Using the spot assay method, 10 μL of phage stock solution at a concentration of approximately 10⁹ PFU/mL was applied to bacterial lawn plates and incubated overnight at 37°C. The following day, colonies capable of growing within the lysis plaques were selected and inoculated into LB liquid medium. After overnight incubation, bacterial DNA was extracted using the E.Z.N.A. Bacterial DNA Kit (D3350, Omega Bio-Tek), followed by whole-genome sequencing and SNP analysis to identify genomic alterations associated with phage resistance. Whole-genome sequencing was performed on an Illumina HiSeq X Ten platform (Illumina Inc., San Diego, CA, USA), generating 150 bp paired-end reads. The genome of the bacteriophage GRNsp8 was removed, and then the raw sequencing data were processed for quality control using *fastp* v0.20.0. High-quality reads were aligned to the WJ48 reference genome using the BWA aligner. SNPs and small insertions/deletions (indels) were identified using *Snippy* v4.6.0, with low-confidence variants filtered based on the sequencing depth and alignment quality. Variant annotation was performed using *snpEff* (http://snpeff.sourceforge.net/SnpEff.html) to assess the potential genomic impact of the detected mutations.

The sensitivity of the identified mutants to phage infection was evaluated using three approaches: spot assay, the double-layer agar plate method (46), and *in vitro* growth curve analysis. The growth curve assay was performed as follows: bacterial cultures were diluted to a final concentration of 10⁶ CFU/mL and inoculated into 96-well microplates. Phage GRNsp8 was added at a multiplicity of infection (MOI) of 0.01. Cultures were incubated with shaking at 37°C for 18 h using a fully automated microbial growth curve analyzer (Ningbo Scientz Biotechnology Co., Ltd., MGC-200Pro), with OD₆₀₀ measured at 30-min intervals. All experiments were performed in triplicate.

### Phage adsorption assays

An optimized protocol was developed based on the method described by Petty et al. (47). Overnight bacterial cultures were mixed with the phage GRNsp8 in LB broth at an MOI of 0.001. As a control, an equal volume of the phage was added to sterile LB broth. Both mixtures were incubated at 37 °C in a shaking incubator set at 200 rpm. At 7, 15, 30, and 45 min, 100 μL of sample was collected and diluted in 900 μL of phosphate-buffered saline (PBS). The samples were centrifuged at 13,000 × *g* for 5 min. After discarding the pellet, the concentration of unadsorbed phages in the supernatant was measured. Adsorption was expressed as the percentage of free phage normalized to the initial titer. The adsorption rate constant was calculated using the following formula:


(1) $$ln\frac{P_{t}}{P_{0}}=-kBt$$

where *k* is the adsorption rate constant (mL/min), *B* is the concentration of bacterial cells, and *t* is the time interval during which the phage titer decreases from *P*₀ (initial) to *P_t_* (final) (48).

### Gene knockout and complementation

A Δ*btuB* mutant of *Salmonella* WJ48 was generated using the λ-Red homologous recombination system, as described by Datsenko and Wanner (49). Primers were designed to amplify the kanamycin resistance cassette flanked by FRT sites from the plasmid pKD4 via PCR. The PCR product was electroporated into *Salmonella* WJ48 cells containing the pKD46 plasmid. Kanamycin-resistant colonies were selected and sequenced. Verified mutants were subsequently transformed with plasmid pCP20, which expresses FLP recombinase, to excise the integrated kanamycin resistance gene from their genomes. Complementation of *btuB* was performed using the genome of *Salmonella* WJ48 as a template. The *btuB* fragment was amplified through PCR. The resulting product was recovered, purified, digested with restriction enzymes, and cloned into the pSTV28 vector to construct the recombinant expression plasmid pSTV28-*btuB*. The correctly sequenced recombinant plasmid was electroporated into *ΔbtuB*-competent cells. Positive transformants were selected on LB agar plates supplemented with chloramphenicol, and the verified strain was designated WJ48-pSTV28-*btuB*.

### Determination of bacterial fitness

#### Bacterial growth curve analysis

Following activation on LB agar plates, bacterial cultures were transferred into LB liquid medium and diluted with PBS to a final concentration of 10⁹ CFU/mL. A 1% inoculum of this suspension was added to fresh LB broth and incubated at 37°C on a shaking platform at 200 rpm for 50 h. During incubation, samples were collected at regular intervals, every 30 min during the logarithmic growth phase. Serial dilutions were performed, and an appropriate dilution was selected for colony enumeration using the plate-count method. A bacterial growth curve was generated by plotting time on the x-axis and log₁₀(CFU/mL) on the y-axis (50).

#### Biofilm formation assay

Quantitative analysis of biofilm formation was conducted using the crystal violet staining method following the protocol of Christensen et al. (51), with modifications. Overnight bacterial cultures grown in LB broth were diluted to a final concentration of 1 × 10⁶ CFU/mL and inoculated into 96-well microtiter plates at 200 μL per well. Each sample was prepared in six replicates, and an additional six wells containing only LB broth without bacteria served as blank controls. Following static incubation at 37°C for 36 h, the culture media were removed, and the wells were washed thrice with sterile PBS to exclude non-adherent cells and air-dried. Next, 200 μL of methanol was added to each well for fixation for 15 min. Next, the methanol was disposed of and the plates were dried at 37°C in an incubator. Each well was stained with 200 μL of 1% crystal violet solution for 15 min. Excess dye was eliminated by rinsing with PBS, and the plates were air-dried. To solubilize the bound dye, 200 μL of 33% glacial acetic acid was added to each well and left for 30 min. Absorbance was quantified at 595 nm utilizing a microplate reader. The experiment was performed twice, and the mean value of 12 replicate measurements was recorded as the final result.

#### Adhesion to CT26 cells

The murine colon carcinoma cell line CT26 was seeded into 12-well culture plates at a density of 1 × 10⁵ cells per well and incubated at 37°C in a 5% CO₂ atmosphere until approximately 80% confluency was achieved. Phage-resistant mutant strains and the wild-type strain were activated in LB broth and cultured to the logarithmic growth phase (OD_600_ = 0.4-0.6). The bacterial cultures were then centrifuged at 2,348 × *g* for 10 min at 4 °C, resuspended in sterile PBS, and adjusted to a final concentration of 1 × 10⁸ CFU/mL. Bacterial suspensions were added to each well at an MOI of 10:1, followed by a 2-h incubation at 37°C. Following incubation, the medium was aspirated, and the wells were washed thrice with PBS to remove non-adherent bacteria. Subsequently, 1 mL of 0.1% Triton X-100 was added to each well and incubated for 20 min to lyse the mammalian cells. The resulting lysates were subjected to 10-fold serial dilutions, and 100 μL of each appropriate dilution was plated on LB agar plates. After 18 h of incubation at 37°C, CFUs were enumerated. The bacterial adhesion rate (CFU per cell) was calculated using the following formula:


(2) $$Adhesion\;rate\left(CFU/ml\right)=\frac{N\times D\times V}{C}$$

where *N* is number of colonies on the plate, *D* is dilution factor, *V* is volume of the lysate, and *C* is initial number of host cells.

#### *In vitro* competition assay

An *in vitro* competition assay was performed to evaluate the relative fitness of WJ48 and *ΔbtuB*. WJ48 was labeled with red fluorescent protein (RFP). Meanwhile, the *ΔbtuB* was labeled with green fluorescent protein (GFP), allowing for fluorescence-based differentiation during co-culture. The strains WJ48-RFP and *ΔbtuB*-GFP were activated on LB agar plates containing kanamycin and inoculated into LB broth for overnight culture at 37°C with shaking at 200 rpm. The following day, the cultures were centrifuged at 2,348 × *g* for 10 min at 4°C, washed, and resuspended in sterile PBS. The cell density was adjusted to 1 × 10⁸ CFU/mL. Equal volumes of WJ48-RFP and *ΔbtuB*-GFP suspensions were mixed at a 1:1 ratio and inoculated into 10 mL of LB broth. This was followed by incubation at 37°C with shaking for 24 h. Samples were collected at 0, 8, 12, and 24 h. At each time point, cultures were serially diluted and plated on LB agar plates supplemented with kanamycin. After incubation at 37°C for 18 h, colonies expressing RFP or GFP were enumerated to determine the CFU of each strain. The relative fitness was calculated according to the following formula (52–55):


(3) $$W=\frac{\text{log}S1_{t-}\text{log}S1_{0}}{\text{log}S2_{t-}\text{log}S2_{0}}$$

where *S*1*_t_* and *S*1_0_ represent the CFU of the mutant strain at the end and beginning of the competition experiment, respectively. *S*2*_t_* and *S*2_0_ represent the corresponding CFU values of the wild-type strain. When the ratio *W* = 1, it indicates that the mutant and wild-type strains have equal fitness. A value of *W* > 1 indicates that the mutant strain possesses higher fitness than the wild-type strain. Meanwhile, *W* < 1 indicates that the mutant strain exhibits lower fitness than the wild-type strain. All the experiments were performed in biological triplicate, and data are presented as mean ± standard deviation (SD).

### *In vivo* assessment of strain virulence

To evaluate the difference in virulence between the wild-type strain and *btuB*-deficient mutant, the LD₅₀ was determined using the method described by Reed and Muench (56). Fifty 4-week-old SPF-grade male BALB/c mice were randomly assigned to 10 groups (*n* = 5). Five groups were infected with WJ48 and received intraperitoneal injections of bacterial suspensions at concentrations of 10⁹, 10⁸, 10⁷, 10⁶, and 10⁵ CFU. Four groups were infected with the *btuB*-deficient strain, receiving intraperitoneal injections at concentrations of 10⁹, 10⁸, 10⁷, and 10⁶ CFU, respectively. The remaining group served as the control and was injected with PBS. Each mouse was monitored closely for clinical signs and mortality over a 7-day period post-inoculation. Survival rates and times were also recorded. The LD₅₀ was calculated using the Reed–Muench method using the following formula:


(4) $$LD_{50}=\left(\frac{50\%-mortality\;at\;lower\;dose}{mortality\;at\;higher\;dose-mortality\;at\;lower\;dose\;}\right)log\left(dose\;interval\right)\times \;lower\;lethal\;dose$$

### Growth in VB_12_-defective medium

Bacterial growth in VB₁₂-deficient medium (M9 minimal medium) was evaluated by measuring the optical density at 600 nm (OD₆₀₀) using a 96-well microtiter plate. The M9 minimal medium was purchased from Merck. Log-phase cultures were diluted with PBS to an OD₆₀₀ of 0.1 and inoculated into the wells. VB₁₂ was added at final concentrations of 1, 10, 100 nM, and 1 μM, with three technical replicates per concentration. Cultures were incubated at 37°C with shaking for 48 h using an automated microbial growth curve analyzer (MGC-200Pro), and OD₆₀₀ was measured every hour. All experiments were performed in triplicate.

### Detection of mutation frequencies in genes in coevolution

A suspension of wild-type bacteria in the logarithmic growth phase (10⁸ CFU/mL) was co-inoculated with phage GRNsp8 at an MOI of 0.01 into 15 mL of LB broth. The cultures were incubated at 37°C with shaking at 200 rpm. At predetermined time points (24, 48, 72, and 216 h), aliquots of the co-culture were collected and centrifuged at 8,000 × *g* for 5 min. The supernatant was then collected, filtered through a 0.22-μm sterile syringe filter, and purified via ultrafiltration to extract phage genomes using the Viral DNA Kit D3892-01 (Omega Bio-Tek). Whole-genome sequencing was conducted on the Illumina HiSeq sequencing platform with paired-end reads (PE150). The raw sequencing data underwent quality control to yield high-quality reads, which were subsequently aligned to the reference genome using the BWA alignment tool. SNPs were identified using Snippy v4.6.0. The resulting bacterial pellets were resuspended in an equal volume of PBS. To assess the mutation frequency at each time point, the resuspended bacterial cultures were serially diluted, and 100 μL of an appropriate dilution was evenly spread onto LB agar plates containing phage GRNsp8 (10⁸ PFU/mL). After air-drying, the plates were incubated in an inverted position at 37°C. The following day, 96 colonies that had grown on phage-containing plates were randomly selected for PCR amplification and sequencing of glycosyltransferase and *btuB* genes. The obtained sequences were aligned with those of the wild-type strain to identify the genetic mutations. The mutation frequency was calculated using the following formula (44):


(5) $$Mutation\;frequency=\frac{Number\;of\;clones\;with\;gene\;mutations}{Total\;number\;of\;clones}$$

Subsequently, a spot assay was conducted to evaluate phage sensitivity, and clones lacking resistance to GRNsp8 were excluded from further analysis.

### Statistical analysis

Statistical analyses were performed with GraphPad Prism v.7.0. For comparisons between two groups, Student’s *t*-test and non-parametric tests were used for normally and non-normally distributed data, respectively. For multiple comparisons, one-way analysis of variance with Bonferroni post-hoc test was employed to assess significance. Unless otherwise noted, research results are presented as mean ± SD. Statistical significance is denoted by asterisks: * *P* < 0.05, **P < 0.01, and ***P < 0.001.

## Data Availability

The reference genome sequence has been deposited at DDBJ/ENA/GenBank under the accession numbers JBSORM000000000, PRJNA1370278, and SAMN53423342 for Genome, Bioproject, and Biosample, respectively.

## SUPPLEMENTAL MATERIAL

Fig. S1 jvi.01950-25-s0001.docx Comparison of BtuB protein structures in mutant and wild-type strains.

ASM does not own the copyrights to Supplemental Material that may be linked to, or accessed through, an article. The authors have granted ASM a non-exclusive, world-wide license to publish the Supplemental Material files. Please contact the corresponding author directly for reuse.
