# Supplementary material for: Fitness costs of phage-driven resistance mutations in Salmonella Enteritidis populations
Source: J Virol. 2026 Jan 16;100(2):e01950-25. doi: 10.1128/jvi.01950-25 (PMC12911894; doi:10.1128/jvi.01950-25)
Supplement: Fig. S1 — Comparison of BtuB protein structures in mutant and wild-type strains. [file jvi.01950-25-s0001.docx]

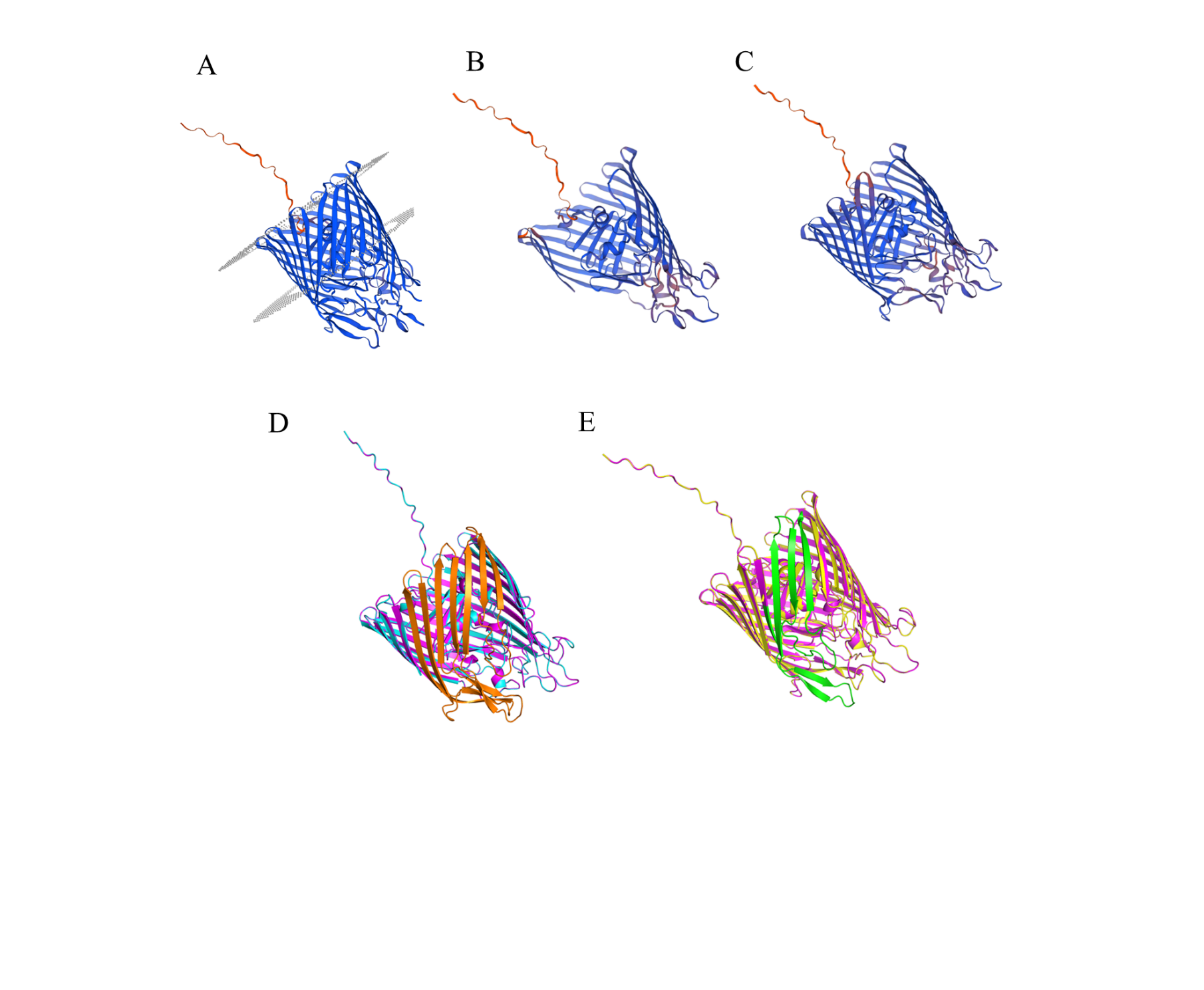


Figure S1. Cartoon representations of BtuB in: A. wild-type, B. VP82, and C. VP84. D. Comparison of the protein structure of BtuB in VP82 and wild-type, with the orange section representing the regions deleted in the mutant. E. Comparison of the protein structure BtuB in VP84 and wild-type, with the green section representing the regions deleted in the mutant.
